# 630 nm LED phototherapy enhances ovarian function and fertility potential in advanced reproductive age females

**DOI:** 10.1002/btm2.70117

**Published:** 2026-02-20

**Authors:** Tiantian Su, Yanling Wan, Fang Fang, Ziyu Li, Cheng Cheng, Jiajia Ai, Nannan Huang, Rong Liang, Jingrong Song, Xiaowei Li, Jiangen Xu, Jianliu Wang, Li Tian

**Affiliations:** ^1^ Department of Obstetrics and Gynecology Peking University People's Hospital Beijing China; ^2^ Reproductive Medical Center Peking University People's Hospital Beijing China; ^3^ Suzhou Institute of Biomedical Engineering and Technology Chinese Academy of Sciences Suzhou China

**Keywords:** 630 nm LED phototherapy, mitochondrial function, oocyte quality, ovarian aging, SDHB

## Abstract

Ovarian aging, a major contributor to declining fertility in women of advanced reproductive age (ARA), is strongly associated with impaired mitochondrial function within oocytes. Although current mitochondrial‐targeted strategies can improve ovarian function, their clinical application remains limited by cost and side effects. In this study, 630 nm light‐emitting diode (LED) phototherapy is shown to ameliorate ovarian aging phenotypes in ARA mice by enhancing mitochondrial complex II activity via upregulation of succinate dehydrogenase subunit B (SDHB). This intervention restores oocyte adenosine triphosphate (ATP) production, improves meiotic progression, and significantly increases blastocyst formation. To assess translational feasibility, a wearable 630 nm LED phototherapy device is developed and evaluated in a pilot clinical study in women with diminished ovarian reserve (DOR). After treatment, the antral follicle count (AFC) significantly increases from a median of 2 (IQR 1–5) to 6 (IQR 2–7; *p* = 0.011), and the number of oocytes retrieved showed a non‐significant tendency to increase from 2 (IQR 1–5) to 5 (IQR 2–7; *p* = 0.083), indicating a potential improvement in ovarian reserve. These findings demonstrate that 630 nm LED phototherapy enhances mitochondrial function and oocyte competence, providing a promising non‐invasive strategy to improve fertility in women affected by ovarian aging.

## Graphical abstract

A non‐invasive phototherapy approach using 630 nm LED light is demonstrated to restore mitochondrial function in advanced reproductive age (ARA) mice's ovaries and improve fertility outcomes. Translational feasibility is shown via a wearable device and a pilot clinical study, offering a promising strategy for enhancing reproductive health in diminished ovarian reserve women. (This figure was created in BioRender. W, L. (2026) https://BioRender.com/2fmutgz).

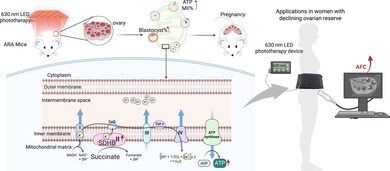

**Translational Impact Statement**

This study introduces a wavelength‐specific wearable LED phototherapy strategy that enhances ovarian function and fertility potential in women with diminished ovarian reserve through a non‐invasive, bioengineered approach. Mechanistically, by targeting ovarian aging via mitochondrial modulation, 630 nm LED exposure improved oocyte quality and reproductive outcomes. Encouraging results from an initial clinical trial support the strong translational potential of this strategy. The safety, affordability, and user‐friendly design of the device underscore its feasibility for broader use, with potential applications in women with declining ovarian reserve.

## 1 INTRODUCTION

Fertility declines in women of advanced reproductive age (ARA), posing a major challenge to reproductive health worldwide.^1^, ^2^ With socioeconomic progress and increasing levels of female education, delayed childbearing is a widespread trend.^3^ However, ovarian function undergoes an irreversible decline with age, characterized by a reduced ovarian reserve, decreased oocyte quality, and decreased reproductive endocrine function. These changes result in lower natural pregnancy rates and an elevated risk of pregnancy failure.^4^, ^5^ Although assisted reproductive technologies (ART) have improved pregnancy outcomes, ARA women continue to face challenges such as low embryo implantation rates and high miscarriage rates.^6^ Current ovulation induction therapies generally do not significantly improve oocyte quality.^7^ Therefore, developing safe and effective interventions to delay ovarian aging and improve oocyte quality has significant clinical and social value for enhancing fertility in ARA women.

A key mechanism underlying ovarian aging is the decline of oocyte mitochondrial function, accompanied by disruptions in energy metabolism. Mitochondrial dysfunction, particularly aberrant electron transport chain (ETC) activity, elevates reactive oxygen species (ROS) levels, impairs ATP synthesis, and can enhance apoptosis, accelerating ovarian senescence.^8^, ^9^ Complex II of the ETC, also known as succinate dehydrogenase (SDH), plays a crucial role in the tricarboxylic acid (TCA) cycle and energy metabolism. A decline in its activity is closely associated with mitochondrial dysfunction in aged ovaries.^10^, ^11^ SDHB, the core SDH subunit, is essential for catalyzing succinate oxidation and transferring electrons to ubiquinone. When SDHB malfunctions, it reduces the efficiency of the ETC and triggers oxidative stress through succinate accumulation and elevated ROS levels, which ultimately impair oocyte quality and embryonic development.^12^, ^13^, ^14^ While stem cell therapy and antioxidants like coenzyme Q10 and resveratrol have shown promise in improving mitochondrial function, their clinical use is limited due to bioavailability, cost, and side effects.^15^, ^16^, ^17^, ^18^

Light‐emitting diode (LED) phototherapy is a non‐invasive physical therapy with minimal side effects, showing promising applications in tissue repair and anti‐aging.^19^ Research suggests phototherapy improves cellular function by modulating intracellular signaling pathways, enhancing mitochondrial energy metabolism, and increasing antioxidant capacity. In recent years, LED phototherapy has been increasingly used across various medical fields, including the promotion of wound healing, the amelioration of skin aging, and the alleviation of chronic pain.^20^, ^21^ Emerging evidence suggests that LED phototherapy at different wavelengths can modulate ovarian function, potentially by regulating the secretion of sex hormones. Research demonstrates that green and blue light exposure enhances the expression of vitellogenin and estrogen receptors in fish and poultry species. This enhancement is linked to increased gonadal index values and elevated plasma estrogen concentrations, which promote ovarian maturation. Red light exhibits a more pronounced stimulatory effect, accelerating oocyte maturation in yellowtail damselfish and elevating reproductive hormone levels and follicle counts in laying hens. Moreover, red light can induce oxidative stress in fish models, reduce the activity of antioxidant enzymes, and affect the nuclear factor erythroid 2‐related factor signaling pathway.^22^ These studies suggest that LED phototherapy may regulate ovarian function and aging by modulating oxidative stress and antioxidant defense mechanisms.^23^ Notably, different LED wavelengths exert wavelength‐dependent biological effects. Shorter red wavelengths such as 630 nm are generally associated with enhanced cellular metabolic activity and mitochondrial function, whereas longer near‐infrared wavelengths such as 850 nm penetrate deeper tissues and are more commonly linked to anti‐inflammatory and immunomodulatory responses.^24^ Nevertheless, research on the application of LED phototherapy for enhancing ovarian function in ARA women remains limited, necessitating further investigation into its biological mechanisms and potential clinical applications.

Here, we investigate the beneficial effects of 630 nm LED phototherapy on ovarian aging and its potential mechanisms, focusing particularly on the regulatory effects of this specific wavelength on mitochondrial function in oocytes. Our results demonstrate that 630 nm LED phototherapy increased the expression levels of ovarian SDHB, enhanced the activity of SDH, optimized the function of the ETC, increased ATP production, improved the quality of oocytes, and reduced ovarian aging. Additionally, we explored the clinical feasibility of a novel wearable LED phototherapy device. We found preliminary evidence suggesting that LED phototherapy partially improved antral follicle counts and oocyte quality in ARA women. Our study addresses a knowledge gap in applying LED phototherapy for ovarian aging interventions and provides support for an innovative strategy to improve reproductive health in ARA women.

## 2 RESULTS

### 2.1 A multispectral LED phototherapy device for mouse experiments

**FIGURE 1:**
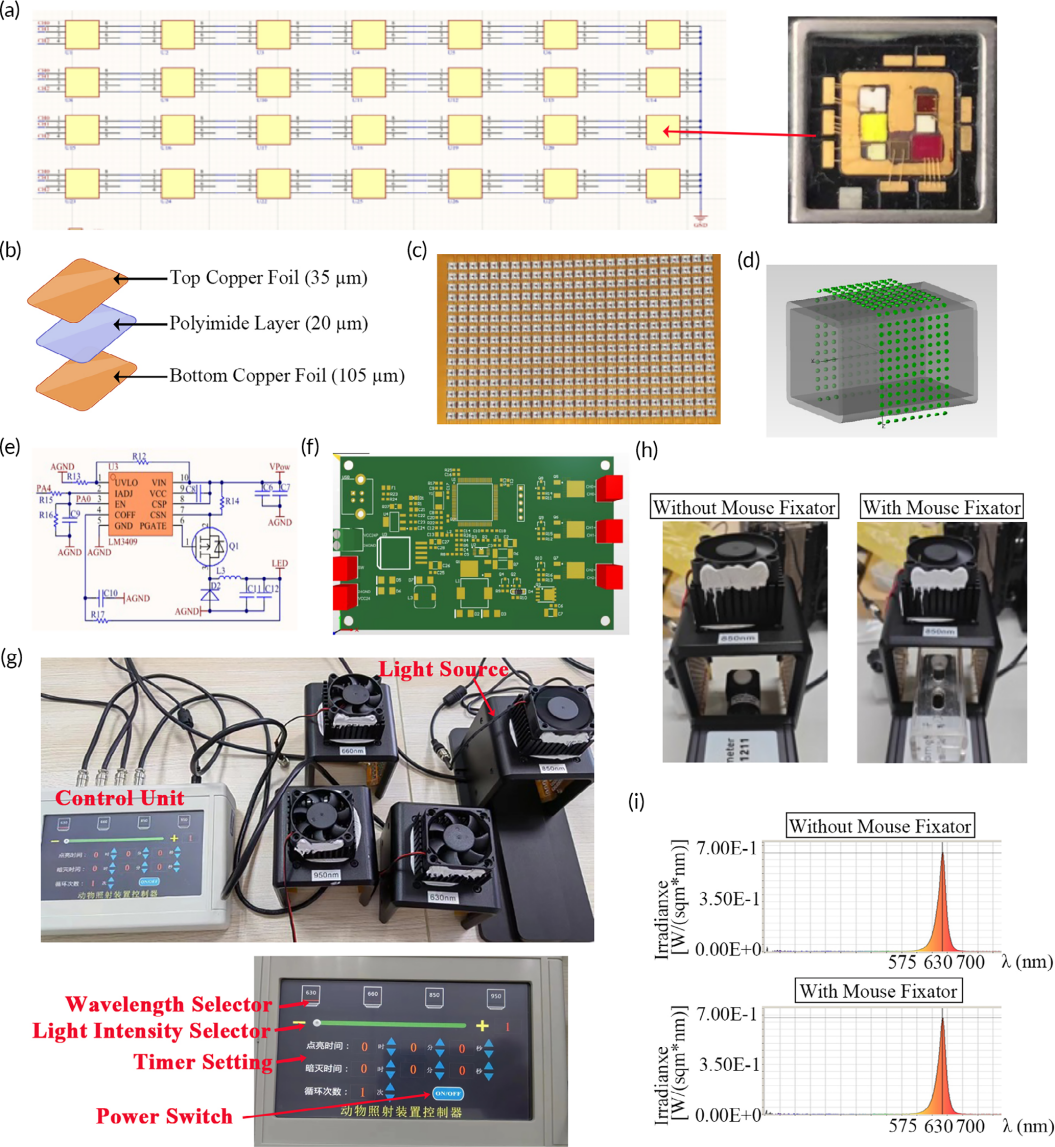
Multispectral LED phototherapy device for mouse experiments. (a) Schematic of the chip‐on‐board packaged LED array. The inset details the multispectral LED chip structure and wire bonding. (b) Cross‐section schematic of the three‐layer flexible polyimide substrate indicating copper foil and dielectric layers with thicknesses. (c) Photograph of the assembled multispectral LED array encapsulated on the flexible circuit substrate. (d) 3D model illustrating the engineered circular array design (9 × 37 LEDs) for uniform illumination. (e) Circuit schematic of the LM3409 constant‐current LED driver module. (f) PCB layout of the main controller module featuring the STM32F103 microprocessor. (g) Photograph of the complete experimental system, including the control unit with touch screen interface and multiple LED light source heads with cooling fans. (h) Schematic illustrating the impact of the fixture on LED light wavelengths using a spectrum analyzer. (i) Representative spectral analysis result showing the irradiance spectrum of the 630 nm channel measured with and without the mouse fixation device.

To investigate the effects of LED light on ovarian function in ARA mice, we independently designed and developed a multispectral LED irradiation device characterized by high luminous efficiency, integration, and thermal stability. This device utilized chip‐on‐board packaging technology, in which LED chips of four distinct wavelengths (630, 650, 850, and 950 nm) were directly bonded to a ceramic substrate using highly conductive silver paste, with electrical interconnections achieved through gold wire bonding (Figure 1a). The ceramic substrate exhibits superior thermal conductivity (~170 W/m K) and coefficient of thermal expansion matching (~4.5 ppm/°C), significantly enhancing heat dissipation and packaging reliability. The LED module is physically protected by glass encapsulation, ensuring its stability and durability during prolonged operation.

The LEDs were subsequently attached to a three‐layer flexible polyimide substrate via reflow soldering. This substrate consists of a 35 μm copper foil top layer etched with circuit patterns, a 20 μm polyimide dielectric layer, and a 105 μm copper foil base layer (Figure 1b). The assembled flexible circuit board (Figure 1c) was attached to an aluminum frame, providing both structural support and heat dissipation. The final LED light source contained a circular array of 9 × 37 LEDs, specifically engineered using a 3D model (Figure 1d) to deliver uniform illumination to the dorsal region of mice near the ovaries. Each LED could achieve a maximum light intensity of 100 mW/cm^2^, rendering it suitable for deep tissue irradiation applications.

Regarding circuit design, the LED array was configured in a seven‐series, four‐parallel arrangement, operating at ~16 V with a constant current of 400 mA. The LED constant current driver module used the LM3409 chip (Texas Instruments), selected for its suitability for high‐power LEDs and stable constant‐current control via its COFT logic structure (Figure 1e).

The complete system integrated a main controller module (Figure 1f), the constant‐current driver module, a communication interface, and a power module. At its core, an STM32F103 microprocessor, paired with an industrial‐grade touch screen, facilitated independent control of multiple LED channels. This design allowed for precisely customizing wavelength combinations, light intensity, and exposure duration, which ensured consistent and reproducible illumination parameters (Figure 1g).

To assess the impact of the fixation device on spectral output before animal experiments, we used a spectral analyzer to measure the spectral distribution of the LED light source before and after passing through the fixture (Figure 1h). The results showed no detectable shifts in peak wavelength or changes in the spectral profile for the selected channels (e.g., 630 nm shown in Figure 1i), indicating that the fixture material did not interfere with the light output. This ensured spectral purity and reproducibility for subsequent animal studies.

### 2.2 630 nm LED phototherapy enhances fertility and mitigates ovarian aging in ARA mice

**FIGURE 2:**
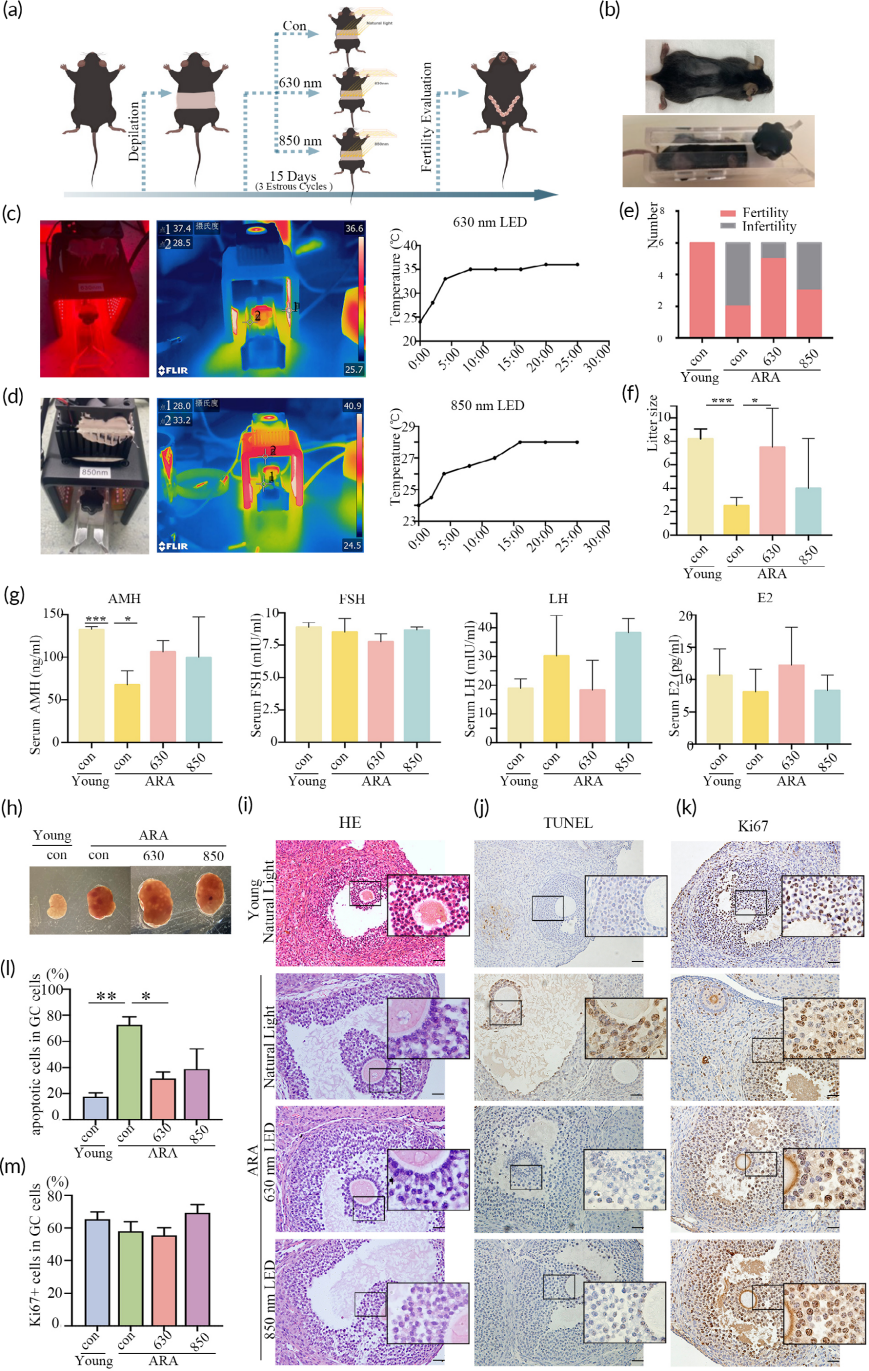
The 630 nm LED phototherapy enhances fertility and mitigates ovarian aging in ARA mice. (a) Schematic illustration of the LED phototherapy experiment in mice. (b) Representative image of dorsal hair removal and positioning of mice within the LED exposure apparatus. (c) Thermal monitoring during 630 nm LED exposure. Representative visible and infrared thermal images (left) and corresponding fixture temperature profile over 30 min (right). (d) Visible and infrared thermal images following 850 nm LED exposure, alongside the associated temperature curve. (e) Comparison of pregnancy rates between young control mice, untreated ARA mice, and ARA mice treated with 630 or 850 nm LED phototherapy (*n* = 6 per group). (f) Average litter size per pregnant female for young control, untreated ARA, 630 nm ARA, and 850 nm ARA groups (*n* = 6 per group). (g) Serum hormone levels were measured in young control, untreated ARA, 630 nm ARA, and 850 nm ARA mice post‐treatment (*n* = 6 per group). (h) Ovarian response to PMSG stimulation after phototherapy in young control, untreated ARA, 630 nm ARA, and 850 nm ARA mice post‐treatment. (i–k) Histological analysis of ovarian morphology, apoptosis (TUNEL assay), and cell proliferation (Ki67 staining) after LED phototherapy. Scale bars: 50 μm. (l) Quantification of the percentage of TUNEL‐positive granulosa cells in antral follicles across treatment groups. (m) Quantification of the percentage of Ki67‐positive granulosa cells in antral follicles across treatment groups. Experiments were independently repeated at least three times. The data are presented as means ± SEM. Statistical significance between two groups was determined using a two‐tailed unpaired Student's *t*‐test. For comparisons involving three or more groups, one‐way analysis of variance (ANOVA) followed by Tukey's post hoc test was applied. *p < 0.05, **p < 0.01, ***p < 0.001.

We investigated the impact of LED phototherapy at various wavelengths on the fertility of ARA mice. Following a literature review, a light intensity of 50 mW/cm^2^ was selected. Eight‐month‐old mice were irradiated daily for 5 min over 15 days, approximating three estrous cycles, to simulate three clinical treatment courses. Fertility assessments were subsequently conducted (Figure 2a). Before phototherapy, the dorsal region near the ovaries of the mice was shaved to minimize interference from fur. During the treatment, the mice were secured and exposed to light at the wavelength, power density, and exposure time shown in Figure 2b.

Thermal stability during irradiation was monitored. Under 630 nm LED phototherapy, the light source emitted a distinct red light. Initial temperature measurements using an infrared thermal imager recorded the light source at 37.4°C and the fixture at 28.5°C. Continuous monitoring over 30 min revealed that the fixture temperature increased, stabilizing at ~35°C after 10 min (Figure 2c). For 850 nm LED phototherapy, the near‐infrared light was invisible to the naked eye. However, the operational status of the light source was observable. The initial light source temperature was recorded at 35.4°C. Over 30 min of continuous operation, the fixture temperature rose, stabilizing at 28°C after ~15 min (Figure 2d).

After LED phototherapy, fertility was evaluated in ARA and young control mice by assessing pregnancy rates and litter sizes. Untreated ARA mice exhibited significantly reduced pregnancy rates and litter sizes compared with young controls. However, following 630 nm LED phototherapy, treated ARA mice showed significantly increased pregnancy rates and litter sizes relative to untreated ARA mice. However, these metrics remained below those observed in the young controls. In contrast, the pregnancy rate and average litter size of ARA mice receiving 850 nm LED phototherapy were comparable to those of untreated ARA controls, indicating no significant improvement (Figure 2e,f). Hormone assays revealed that serum Anti‐Müllerian Hormone (AMH) levels were significantly lower in untreated ARA mice than in young controls. After 630 nm LED phototherapy, AMH levels in ARA mice were partially restored, while follicle‐stimulating hormone (FSH), luteinizing hormone (LH), and estradiol (E2) levels remained unchanged compared with untreated ARA controls. In contrast, 850 nm LED phototherapy did not significantly alter the AMH, FSH, LH, or E2 levels in aged mice (Figure 2g).

To minimize the effects of the oestrous cycle and to assess the ovarian responsiveness to hormonal stimulation following LED phototherapy, ARA mice were administered Pregnant Mare Serum Gonadotropin (PMSG), and ovarian tissues were collected 46 h post‐injection for histological evaluation. Morphological analysis revealed a significantly increased number of antral follicles in the ovaries of mice in the 630 nm LED phototherapy, compared with the untreated ARA control and 850 nm LED phototherapy groups (Figure 2h). Hematoxylin and eosin (H&E) staining showed no apparent morphological changes in the ovaries due to either 630 or 850 nm LED phototherapy wavelength (Figure 2i). Terminal deoxynucleotidyl transferase dUTP nick end labelling (TUNEL) staining indicated a significantly higher level of apoptosis among granulosa cells within the antral follicles of untreated ARA mice relative to their younger counterparts. However, 630 nm LED light treatment significantly attenuated this apoptosis (Figure 2j,l). In contrast, immunohistochemical staining for the proliferation marker Ki67 showed that LED exposure did not significantly alter the number of Ki67‐positive granulosa cells in these follicles (Figure 2k,m).

Collectively, these results suggest that exposure to 630 nm LED phototherapy improved fertility outcomes, including pregnancy rate and litter size, partially restored AMH levels, increased antral follicle count following stimulation, and reduced granulosa cell apoptosis in ARA mice, whereas 850 nm LED phototherapy had a limited impact. Subsequent investigations will aim to delineate the molecular pathways underlying the effects of 630 nm LED phototherapy on ovarian function.

### 2.3 630 nm LED phototherapy alters ovarian cellular composition toward a younger profile in ARA mice

**FIGURE 3:**
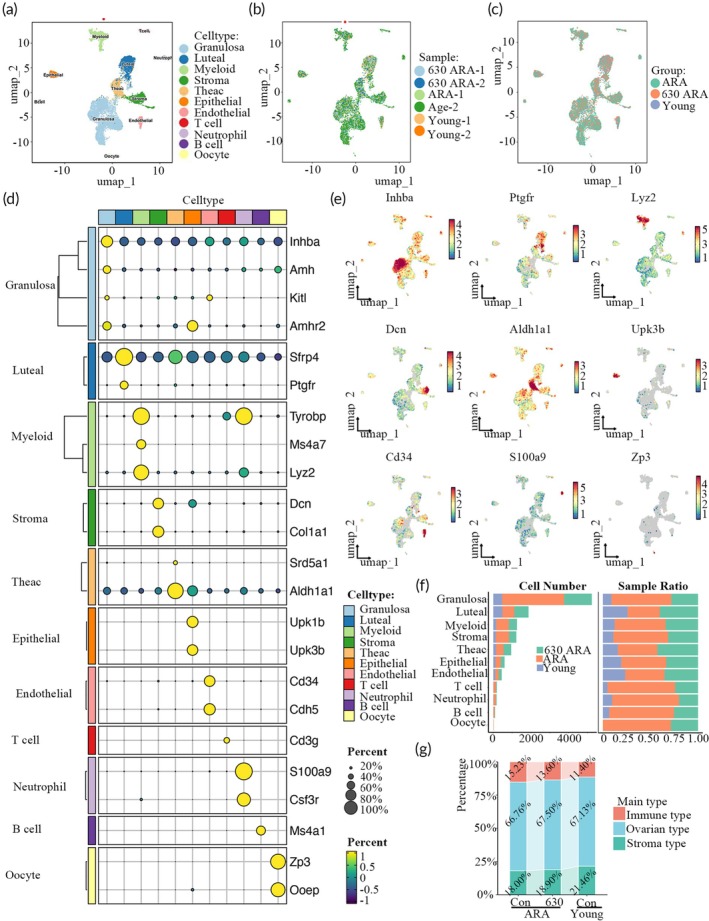
The 630 nm LED phototherapy alters ovarian cellular composition toward a younger profile in ARA mice. (a) UMAP plot of single‐cell RNA sequencing data from mouse ovarian tissue, identifying 11 distinct cell types. (b) UMAP plot displaying the distribution of cells from individual samples. (c) UMAP plot illustrating cell distribution across different experimental groups. (d) Dot plot showing the expression of canonical marker genes across identified cell types; dot size represents the percentage of cells expressing each gene, while color intensity indicates the average expression level. (e) Feature plot mapping the expression of representative canonical marker genes onto the UMAP projection; color gradient reflects expression levels. (f) Bar plot depicting each cell type's abundance and proportional distribution across experimental groups, with colors distinguishing the groups. (g) The 11 identified cell types were classified into three major categories—immune, ovarian, and stromal cells—and their proportional changes across experimental groups were shown. *n* = 2 per group for single‐cell RNA sequence.

Single‐cell RNA sequencing (scRNA‐seq) was used to generate a cellular atlas of ovarian tissues from three groups: young control mice (Young), untreated ARA mice, and ARA mice receiving 630 nm LED phototherapy (630). Uniform Manifold Approximation and Projection (UMAP) visualization of cell clustering enabled the identification of 11 distinct cell populations, including oocytes, granulosa cells, stromal cells, and various immune cell subsets (Figure 3a). The distribution of cells originating from each sample and experimental group within the UMAP space is shown in Figure 3b,c, respectively. The expression profiles of established marker genes across the identified cell types were visualized using DotPlot analysis (Figure 3d,e), confirming accurate cell‐type annotation.

We analyzed the abundance and distribution of various ovarian cell types across the groups to assess the impact of 630 nm LED phototherapy on ovarian cellular composition associated with aging. Compared with untreated ARA mice, the 630 nm ARA group exhibited a directional shift in the relative proportions of multiple ovarian cell types toward a young‐like cellular composition (Figure 3f). This compositional tendency suggests that LED phototherapy may partially mitigate cellular alterations associated with ovarian aging in ARA mice. Furthermore, when the 11 identified cell types were categorized into the broader functional groups of immune, ovarian, and stromal cells, consistent directional changes toward a young‐like distribution were observed across these categories in the 630 nm ARA group relative to the untreated ARA group (Figure 3g). To provide quantitative detail, the percentage composition of major ovarian cell types across the three groups is summarized in Figure S2.

### 2.4 630 nm LED phototherapy modulates ovarian metabolism in ARA mice

**FIGURE 4:**
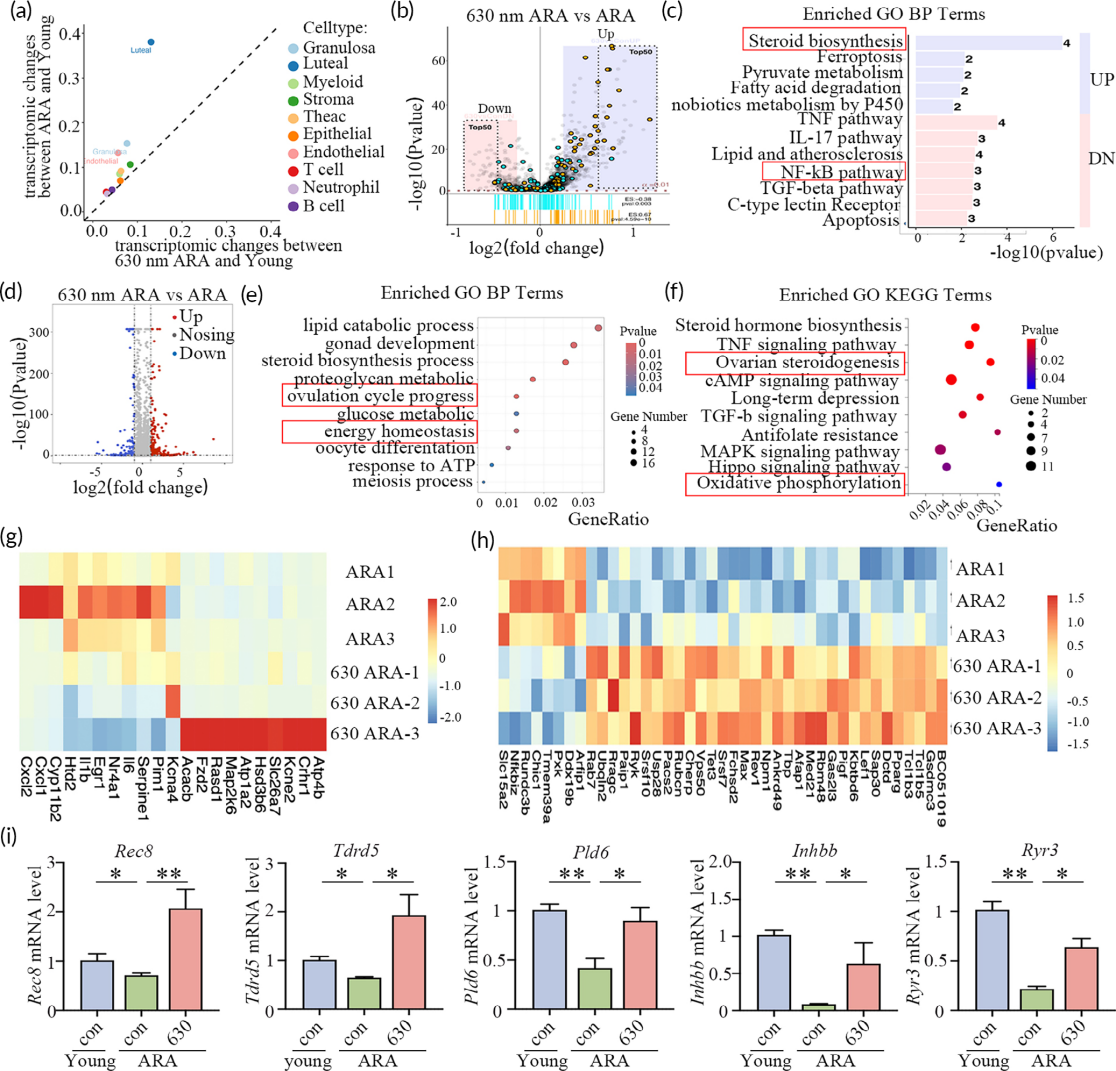
The 630 nm LED phototherapy regulates ovarian metabolism in ARA mice. (a) Scatter plot comparing transcriptional changes across ovarian cell types from scRNA‐seq data. The *x*‐ and *y*‐axes represent the magnitude of transcriptional changes in the 630 nm ARA and untreated ARA groups, respectively, relative to the young control group. (b) Volcano plot displaying differential gene expression between the 630 nm ARA and untreated ARA ovarian cells based on scRNA‐seq analysis. (c) GO enrichment analysis of differentially expressed genes identified via scRNA‐seq between the 630 nm ARA group and the untreated ARA group. (d) Volcano plot of bulk RNA sequencing analyses performed on ovarian tissues from the 630 nm ARA group versus the untreated ARA group. Genes exhibiting ≥2‐fold expression changes (*p* < 0.05) are highlighted, with downregulated genes indicated in blue and upregulated genes in orange. (e) GO biological process enrichment analysis of differentially expressed genes identified from bulk RNA‐seq modulated by LED treatment. (f) KEGG pathway enrichment analysis of genes responsive to 630 nm LED phototherapy. (g) Heatmap demonstrating the effect of 630 nm LED phototherapy on the expression of genes implicated in energy metabolism. The color scale from blue to red represents low to high expression, respectively. (h) Heatmap illustrating changes in mitochondrial function‐related gene expression following LED phototherapy. The color scale from blue to red represents low to high expression, respectively. Columns represent individual samples from treatment groups. *n* = 2 per group for single‐cell RNA sequence. *n* = 3 per group for bulk RNA‐sequence. (i) RT‐qPCR validation of relative mRNA expression levels for selected mitochondrial function‐related genes (*Rec8*, *Tdrd5*, *Pld6*, *Inhbb*, *Ryr3*) in ovarian tissues from young control, untreated ARA, and 630 nm ARA mice. *Gapdh* was used as an internal reference. Experiments were independently repeated at least three times. The data are presented as means ± SEM. The statistical significance between two groups was determined using a two‐tailed unpaired Student's *t*‐test. For comparisons involving three or more groups, one‐way ANOVA followed by Tukey's post hoc test was applied. *p < 0.05, **p < 0.01, ***p < 0.001.

We performed single‐cell transcriptome analysis to investigate the molecular mechanisms underlying the improvement of ovarian function in ARA mice by 630 nm LED phototherapy. The results revealed that, following 630 nm LED light intervention, the transcript levels in various ovarian cell types of ARA mice shifted to resemble those observed in the young control group, compared with the untreated ARA group (Figure 4a). Differential gene expression analysis (Figure 4b) and Gene Ontology (GO) enrichment analysis indicated that genes associated with hormone synthesis were upregulated in the 630 nm ARA group and those linked to oxidative stress were downregulated (Figure 4c).

To elucidate further the mechanisms underlying the effects of 630 nm LED phototherapy, bulk RNA sequencing was conducted on ovarian tissues from 630 nm‐treated ARA mice and untreated ARA mice, revealing 333 significantly differentially expressed genes (Figure 4d). GO enrichment analysis for biological processes indicated that these differentially expressed genes are involved in key processes such as gonadal development, follicular cycling, and energy metabolism, aligning with the observed phenotypic improvements (Figure 4e). KEGG pathway analysis further demonstrated that 630 nm LED phototherapy influences signaling pathways including hormone synthesis, oxidative phosphorylation, and TGF‐β pathways (Figure 4f). Notably, significant alterations were observed in genes associated with energy metabolism (Figure 4g) and mitochondrial function (Figure 4h). Furthermore, we validated the expression of key mitochondrial function‐related genes using RT‐qPCR. The results showed that the 630 nm LED phototherapy significantly increased the ovarian mRNA levels of *Rec8*, *Tdrd5*, *Pld6*, *Inhbb*, and *Ryr3* in ARA mice, bringing their expression closer to levels observed in the young control group (Figure 4i). Collectively, these transcriptomic findings indicate that 630 nm LED phototherapy influenced mitochondrial function and energy metabolism in the ovaries of ARA mice, suggesting a potential mechanism underlying its rejuvenating effects.

### 2.5 630 nm LED phototherapy enhances mitochondrial function in the ovaries of ARA mice via upregulation of SDHB

**FIGURE 5:**
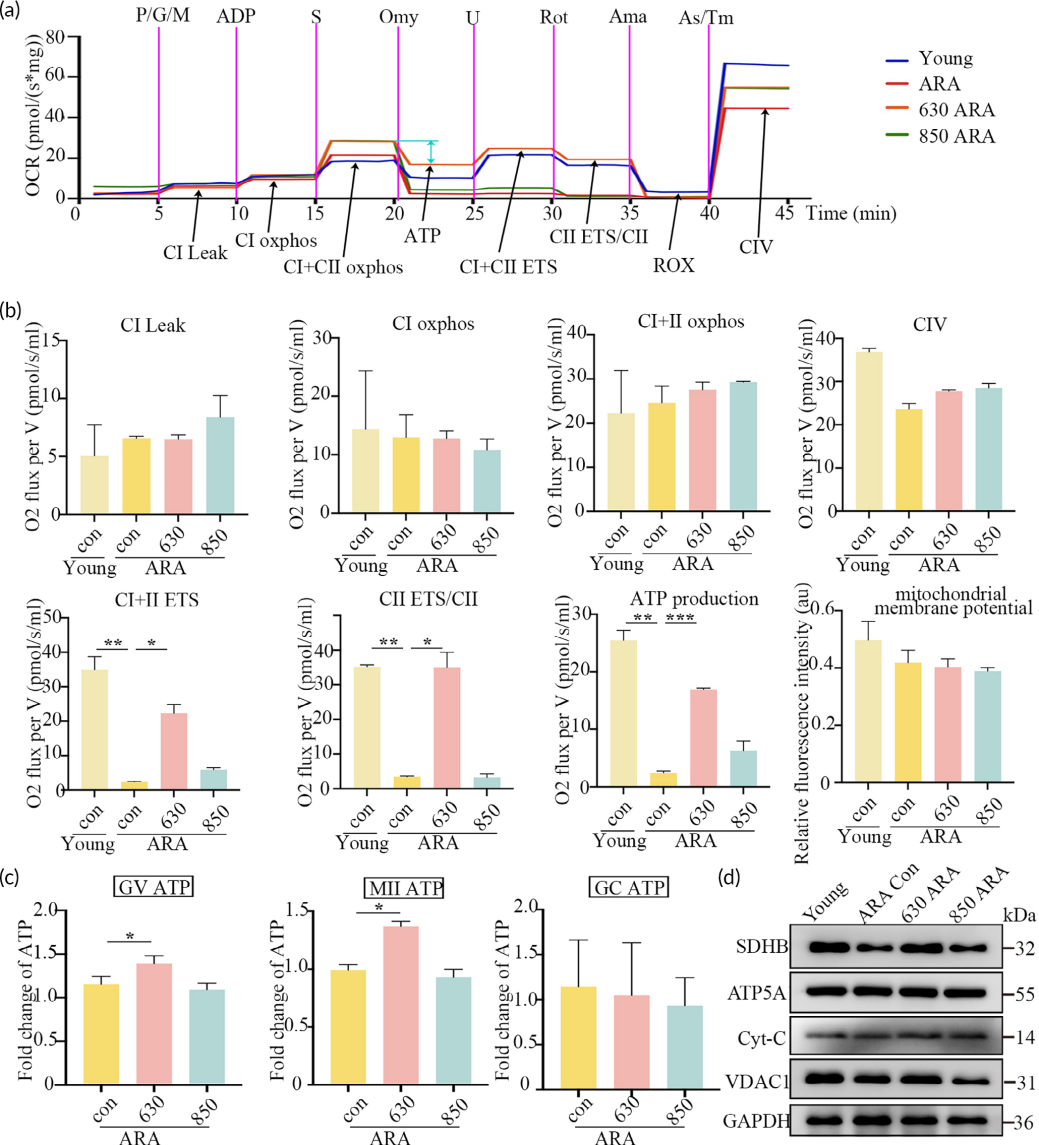
The 630 nm LED phototherapy enhances mitochondrial function in the ovaries of ARA mice via upregulation of SDHB. (a) Representative real‐time oxygen concentration and consumption rate measurements from ovarian tissue homogenates obtained using Oxygraph‐2k respirometry. Measurement points indicating specific mitochondrial respiratory parameters are labeled. (b) Quantification of mitochondrial complex activities derived from respirometry data during ATP production. *n* = 3 per group. (c) ATP levels in GV oocytes, MII oocytes, and granulosa cells from the indicated groups, assessed by ATP quantification assays. (d) Western blot analysis of key proteins associated with mitochondrial function. All experiments were independently repeated at least three times. The data are presented as means ± SEM. Statistical significance between two groups was determined using a two‐tailed unpaired Student's *t*‐test. For comparisons involving three or more groups, one‐way ANOVA followed by Tukey's post hoc test was applied. *p < 0.05, **p < 0.01, ***p < 0.001. ATP production, ATP synthesis capacity; C IV, activity of complex IV; CI Leak, proton pumping capacity of complex I; CI OXPHOS, oxidative phosphorylation capacity of complex I; CI + CII ETS, maximal electron transfer capacity of complexes I and II; CI + CII OXPHOS, combined oxidative phosphorylation capacity of complexes I and II; CII ETS, respiratory capacity of complex II; ROX, non‐mitochondrial oxygen consumption.

To elucidate how 630 nm LED phototherapy improves mitochondrial function in the ovaries of ARA mice, mitochondrial respiration was evaluated using the Oxygraph‐2k respirometry system. Figure 5a depicts representative real‐time measurements of oxygen concentration and consumption rates under different substrate/inhibitor conditions. Based on the oxygen consumption data, we calculated the activity of individual mitochondrial complexes. The results demonstrated that 630 nm LED phototherapy significantly increased the activity of mitochondrial complex II and ATP production, whereas the activity of mitochondrial complexes I, III, IV, and mitochondrial membrane potential remained unchanged compared with untreated ARA controls (Figure 5b). Direct ATP quantification assays indicated that 630 nm LED phototherapy significantly elevated ATP levels in germinal vesicle (GV) and metaphase II (MII) oocytes from ARA mice but had no detectable effect on granulosa cells (Figure 5c). Western blot analysis of mitochondrial proteins demonstrated reduced levels of the complex II subunit SDHB in ovaries from untreated ARA mice relative to the young control group. Notably, 630 nm LED phototherapy significantly restored SDHB protein levels in ARA mice, while the levels of ATP5A1 (complex V subunit), Cyt‐C (electron carrier), and VDAC1 (mitochondrial outer membrane protein) remained unaffected by treatment or age (Figure 5d).

Collectively, these results demonstrate that 630 nm LED phototherapy enhanced ovarian energy metabolism and function primarily through modulation of mitochondrial complex II activity in oocytes, improving overall ovarian function. These findings indicate a mechanism by which 630 nm LED phototherapy reduces ovarian aging, highlighting its potential as a non‐invasive intervention to enhance fertility in ARA individuals.

### 2.6 630 nm LED phototherapy enhances oocyte quality in ARA mice

**FIGURE 6:**
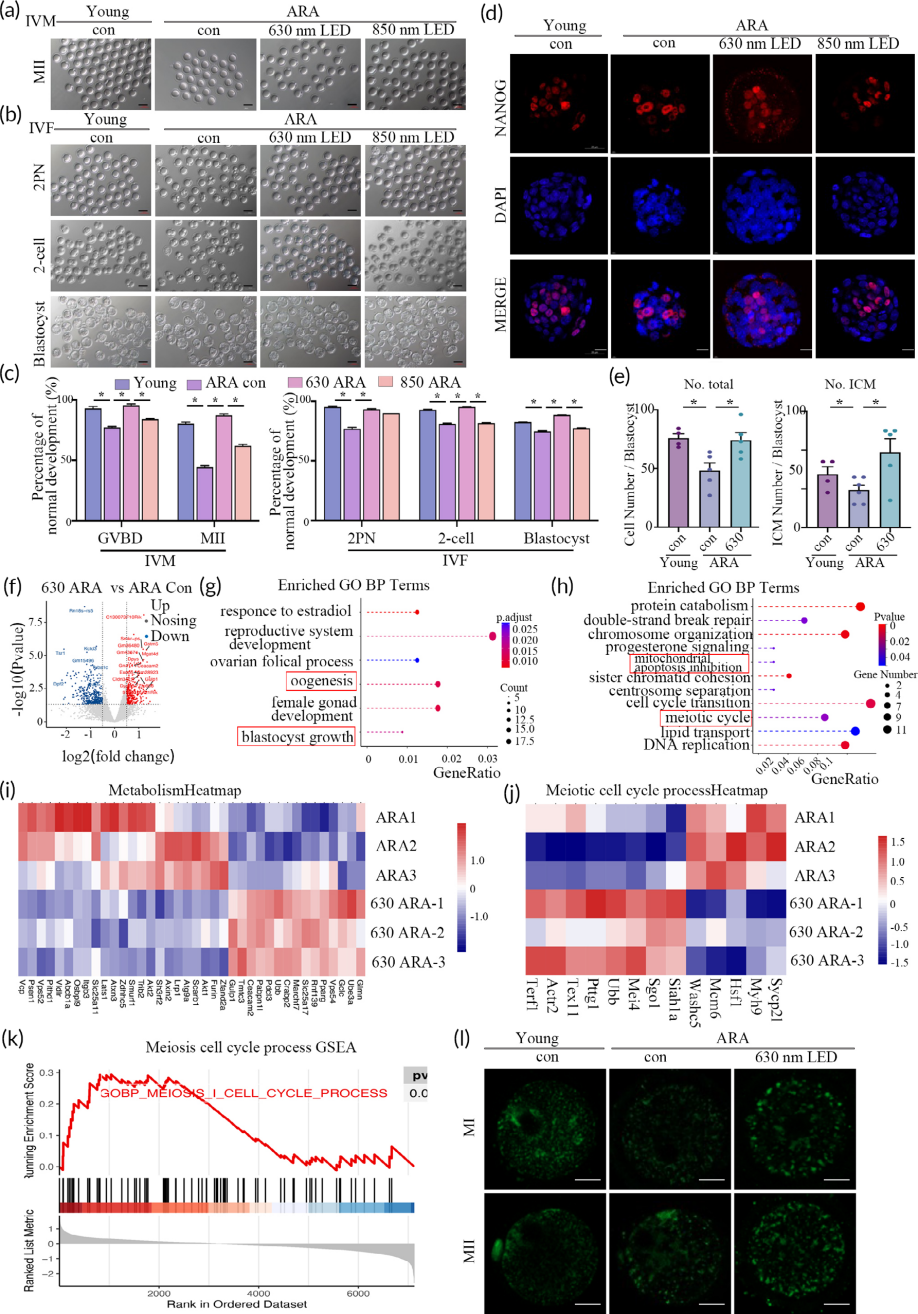
The 630 nm LED phototherapy enhances oocyte quality in ARA mice. (a) Effects of LED phototherapy on metaphase II (MII) rates evaluated by IVM. Scale bar: 100 μm. *n* = 6 per group. (b) Impact of LED phototherapy on embryo development assessed by IVF. Scale bar: 100 μm. (c) Statistical comparison of germinal vesicle breakdown (GVBD) and MII rates (left panel), 2PN, 2‐cell, and blastocyst rates (right panel) following LED phototherapy for the indicated groups. *n* = 6 per group. (d) Representative images of blastocysts from young control, untreated ARA, and 630 nm ARA mice. The ICM was stained with NANOG (red), and chromosomes were counterstained with DAPI (blue). Scale bar: 20 μm. (e) Quantification of the total cell number and ICM cell number in blastocysts derived from young control, untreated ARA, and 630 nm ARA mice. (f) Volcano plot illustrating differentially expressed genes identified by Smart‐seq RNA sequencing of GV oocytes, comparing 630 nm ARA versus untreated ARA mice. *n* = 3 per group. (g) GO enrichment analysis for biological processes of the differentially expressed genes identified in (f). (h) GO enrichment analysis of the differentially expressed genes identified in (f), highlighting key altered signaling pathways. (i) Heatmap illustrating expression patterns of differentially expressed genes associated with mitochondrial function (identified in f) in GV oocytes. (j) Heatmap illustrating expression patterns of differentially expressed genes associated with meiosis (identified in f) in GV oocytes. (k) GSEA plot indicating enrichment of meiosis‐related gene sets, based on the comparison established in (f). (l) Mitotracker staining was used to evaluate differences in mitochondrial distribution in oocytes. Scale bar: 20 μm. All experiments were independently repeated at least three times. The data are presented as means ± SEM. Statistical significance between two groups was determined using a two‐tailed unpaired Student's *t*‐test. For comparisons involving three or more groups, one‐way ANOVA followed by Tukey's post hoc test was applied. *p < 0.05, **p < 0.01, ***p < 0.001.

To investigate further the specific role of LED phototherapy in improving ovarian function, 8‐month‐old mice (ARA mice) and young control mice were used. ARA mice were exposed to LED irradiation (630 nm or 850 nm; intensity 50 mW/cm^2^) for 5 min daily over 15 consecutive days. Oocytes were subsequently collected and cultured in vitro to evaluate maturation potential. Compared with young control mice, untreated ARA mice exhibited significantly lower MII rates, assessed by in vitro maturation (IVM). Notably, 630 nm LED treatment restored MII rates in ARA mice to levels comparable to young control mice (Figure 6a,c). Following in vitro fertilization (IVF), oocytes from untreated ARA mice exhibited reduced 2‐pronuclear (2PN) rates relative to those from young control mice, whereas 630 nm LED exposure significantly enhanced both 2PN and blastocyst formation rates in the ARA group; 850 nm LED treatment had no significant effects (Figure 6b,c). Immunofluorescence labelling for NANOG, an inner cell mass (ICM) marker, indicated improved blastocyst morphology following 630 nm LED treatment, demonstrating higher embryo quality (Figure 6d). Correspondingly, total cell numbers and ICM counts in blastocysts from 630 nm LED‐treated ARA mice were significantly higher than those from untreated ARA mice (Figure 6e). These results collectively indicate that 630 nm LED phototherapy substantially enhanced oocyte quality and developmental potential in aged mice.

To delineate further the molecular mechanisms underlying these improvements, Smart‐seq RNA sequencing was performed on GV oocytes from untreated ARA mice and 630 nm‐treated ARA mice, identifying numerous differentially expressed genes (Figure 6f). Biological process enrichment analysis revealed enrichment of gene sets involved in follicular development, oogenesis, and embryogenesis, consistent with earlier results (Figure 6g). Moreover, signaling pathways implicated in meiosis, mitochondrial function, and chromosome organization were significantly altered by 630 nm LED exposure (Figure 6h). Heatmaps illustrate the expression patterns of genes associated with mitochondrial function (Figure 6i) and meiosis (Figure 6j), with expression levels indicated by a gradient from blue (low) to red (high). Gene set enrichment analysis (GSEA) further confirmed an upregulation of meiosis‐related gene expression following 630 nm LED phototherapy (Figure 6k). Mitotracker staining showed improved mitochondrial distribution in oocytes after 630 nm LED treatment (Figure 6l). These data collectively support that 630 nm LED phototherapy enhances mitochondrial function in oocytes, improving oocyte quality and embryonic developmental competence, suggesting potential applications in enhancing ART outcomes in ARA women.

### 2.7 Wearable 630 nm LED phototherapy as a potential approach to improve fertility outcomes in women with declining ovarian reserves

**FIGURE 7:**
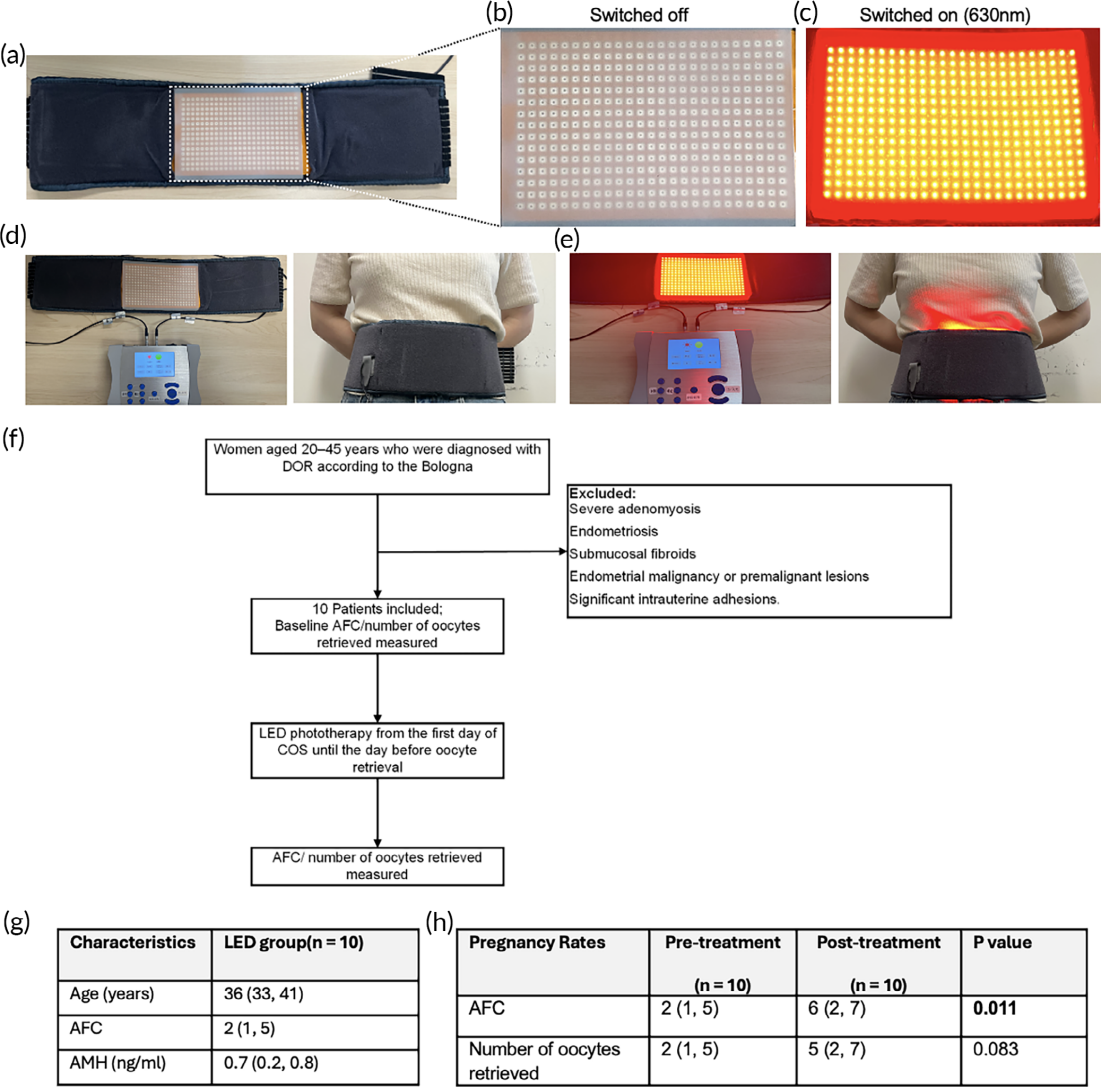
The 630 nm LED phototherapy as a potential approach to improve fertility outcomes in women with declining ovarian reserve. (a) Representative image of the wearable LED device with waistband component designed for use by women. (b) Device shown in the switched‐off state. (c) Device activated, emitting 630 nm LED light. (d) Device worn by the female participant, illustrating the off state. (e) The activated device in use on the female patient, showing illumination of the targeted area. *(f)* Schematic workflow of the clinical protocol for wearable LED phototherapy in patients of DOR. (g) Baseline characteristics of the 10 enrolled women of DOR. (h) Changes in AFC and number of retrieved oocytes before and after 630 nm LED phototherapy in the clinical cohort (*n* = 10 patients), with matched data from the control cohort (*n* = 10 patients).

Women with declining ovarian reserve—typically characterized by reduced AFC and AMH levels and clinically classified under the Bologna criteria for diminished ovarian reserve—represent a population with impaired ovarian responsiveness and limited fertility treatment options. To translate our preclinical findings toward clinical applications, we developed a wearable LED phototherapy device suitable for women's use. The device is a low‐voltage, rechargeable, portable, belt‐like instrument designed for home use. After sterilizing the device with an alcohol wipe, it is applied directly to the skin of the lower abdomen, covering the ovarian area, and secured with a Velcro strap. The device features flexible circuit boards with silicone‐based light diffusion technology, allowing uniform LED illumination at intensities up to 20 mW/cm^2^. To ensure thermal stability during prolonged use, the device incorporates flexible printed circuit boards that increase the heat‐dissipating copper area (Figure 7a–c). While the device can emit multiple wavelengths, the 630 nm wavelength was used in a continuous mode for this clinical observation. The device was set to deliver an energy density of 15 mW/cm^2^ for the 630 nm wavelength, with a daily treatment duration of 30 min. Eligible participants were instructed to use the device daily until the day before oocyte retrieval. The user interface allowed for adjustment of parameters, but the parameters were preset for this study (Figure 7d,e).

In this single‐arm interventional trial, 10 women aged 20–45 years with DOR, diagnosed based on the Bologna criteria, received daily 630 nm LED phototherapy prior to their subsequent IVF/ICSI cycle (Figure 7f). At baseline, the median age was 36 years (IQR 33–41), with a median AFC of 2 (IQR 1–5) and AMH level of 0.7 ng/mL (IQR 0.2–0.8) (Figure 7g). After treatment, AFC significantly increased from a median of 2 (IQR 1–5) to 6 (2–7, *p* = 0.011), and oocyte yield showed a non‐significant tendency to increase from 2 (1–5) to 5 (2–7, *p* = 0.083), suggesting enhanced ovarian reserve (Figure 7h).

A clinical case is presented in Figure S1. This patient was selected because she was the only enrolled participant who had achieved a complete pregnancy outcome at the time of manuscript submission. This 36‐year‐old woman had a baseline AMH level of 0.917 ng/mL and a history of repeated IVF failures. After receiving three courses of 630 nm LED phototherapy, her AMH level rose to 1.410 ng/mL (Figure S1A,B). During her sixth IVF cycle, eight oocytes were retrieved, and one high‐quality blastocyst was obtained (Figure S1C). Although two subsequent embryo transfers were unsuccessful, she subsequently achieved a spontaneous pregnancy, which was ongoing at the time of submission without reported complications (Figure S1D).

These preliminary clinical observations demonstrate measurable improvements in ovarian parameters following 630 nm LED phototherapy. Further randomized controlled studies are needed to evaluate the therapeutic efficacy and generalizability of this approach in women with ovarian insufficiency.

In summary, this study demonstrates that 630 nm LED phototherapy reduced ovarian aging and enhanced fertility in ARA mice through a multi‐step mechanism. Specifically, the phototherapy upregulated SDHB protein levels within the ovary, increasing the activity of mitochondrial complex II (SDH enzyme). This enhancement subsequently improved the efficiency of the mitochondrial ETC, leading to increased ATP levels. These mitochondrial improvements are pivotal in counteracting ovarian aging phenotypes and improving reproductive capacity in these preclinical models. Furthermore, our findings indicated that the beneficial effects of 630 nm LED phototherapy are primarily mediated by enhancing mitochondrial function within oocytes. This targeted action elevates oocyte ATP levels, thereby improving critical parameters such as oocyte MII rates, developmental potential (evidenced by blastocyst rates), which contributed to overall oocyte quality, and the observed mitigation of ovarian aging.

## 3 DISCUSSION

Age‐related ovarian dysfunction, characterized by declining oocyte quality, reduced follicular reserves, and an impaired ovarian microenvironment, remains the primary limiting factor for fertility in ARA women.^25^ As societal shifts increasingly delay childbearing, particularly in developed countries, age‐associated decline in fertility has emerged as a critical health issue.^26^ According to the US Centers for Disease Control and Prevention (CDC, 2020), clinical pregnancy rates from assisted reproductive technologies drop significantly with age: from 68.9% in women younger than 35 years to 58.2% between ages 35 and 37, and precipitously to 7% after age 42.^27^ These statistics highlight an urgent clinical need for effective strategies to counteract ovarian aging.

LED phototherapy, a non‐invasive treatment with minimal side effects, has emerged as a promising approach in reproductive medicine, primarily due to documented cellular effects on signaling pathways, tissue regeneration, and aging processes.^28^, ^29^, ^30^ While beneficial impacts on hormonal balance and oxidative stress have been reported,^31^ systematic evaluation of its effects on ovarian function remains scarce. In this study, we investigated the therapeutic potential of 630 nm LED phototherapy on ovarian aging in a mouse model. Our findings demonstrated that daily exposure (5 min/day for 15 days) to 630 nm, but not 850 nm, significantly increased serum AMH levels, pregnancy rates, and litter sizes in ARA mice (Figure 2). Histological analyses after superovulation showed enhanced numbers of antral follicles, suggesting improved ovarian responsiveness in this group.

Single‐cell transcriptomic profiling demonstrated that the cellular composition of ovaries from 630 nm‐treated ARA mice resembled that of young control mice, indicative of a partial reversal of ovarian aging at the cellular level (Figure 3). The treatment appeared to mitigate typical hallmarks of ovarian aging, including collagen depletion, increased fibrosis, oxidative stress, and impaired granulosa‐oocyte interactions. Although analysis of intercellular interactions was limited by low oocyte recovery, these findings suggest positive modulation of ovarian aging phenotypes by 630 nm LED phototherapy.

Since mitochondrial dysfunction critically contributes to ovarian aging through impaired energy metabolism, oxidative damage, and apoptosis, thus compromising oocyte quality,^9^, ^32^, ^33^ we examined mitochondrial pathways in our study. RNA‐seq analysis revealed that 630 nm LED phototherapy significantly affected genes linked to gonadal development, mitochondrial function, and energy metabolism (Figure 4). Subsequent assays confirmed enhanced mitochondrial complex II activity and elevated ATP levels specifically in the GV and MII oocytes, rather than granulosa cells, indicating targeted mitochondrial improvements in oocytes (Figure 5). SDH acts as a critical interface between the TCA cycle and the electron transport chain (ETC).^34^ Impaired SDH activity disrupts electron transport, reduces ATP synthesis, and promotes succinate accumulation. Elevated succinate levels further inhibit SDH function and enhance ROS generation via reverse electron transfer (RET), exacerbating mitochondrial DNA damage, protein oxidation, and initiating a detrimental oxidative stress loop.^35^, ^36^ Moreover, accumulated succinate can activate inflammatory signaling through its receptor, succinate receptor 1, triggering the release of pro‐inflammatory cytokines such as IL‐1β and TNF‐α, accelerating ovarian inflammation and fibrosis.^37^, ^38^ In oocytes, aberrant succinate metabolism compromises meiotic accuracy and early embryonic development, significantly impairing fertilization and reproductive outcomes.^39^, ^40^ Notably, oocytes lack the ability to use glucose directly and rely predominantly on granulosa cell‐derived pyruvate.^41^, ^42^ Upon entering mitochondria, pyruvate undergoes TCA cycling and oxidative phosphorylation, generating the ATP essential for meiosis and early embryogenesis.^43^ Although our data demonstrate that 630 nm LED phototherapy enhanced ovarian SDH activity in ARA mice, technical constraints precluded the precise identification of the specific ovarian cell types mediating these improvements beyond the observed ATP increase in oocytes. Our findings showed that SDHB protein levels were reduced in aged ovaries but increased following 630 nm LED treatment, consistent with previous reports linking SDHB deficiency to impaired follicular development and oocyte quality as a consequence of mitochondrial dysfunction. These findings highlight that 630 nm phototherapy acts primarily by enhancing mitochondrial energy metabolism in oocytes, which aligns with the core pathophysiology of ovarian aging. In contrast, longer wavelengths such as 850 nm, while capable of deeper tissue penetration, are predominantly associated with anti‐inflammatory or tissue‐repair responses and may be less effective in modulating mitochondrial ATP‐generating pathways within oocytes. This mechanistic distinction provides a plausible explanation for why 630 nm, but not 850 nm, produced significant improvements in ovarian function in our model.

Whether 630 nm LED phototherapy can enhance ART outcomes in ARA women was a central focus of this study. We performed IVM and IVF assays following LED phototherapy and demonstrated that 630 nm LED significantly improved oocyte maturation, fertilization, and blastocyst rates in ARA mice, indicating enhanced oocyte quality and developmental competence (Figure 6). In contrast, 850 nm irradiation had no notable effect on these parameters. Further transcriptomic (Smart‐seq) analyses revealed that 630 nm LED phototherapy affected mitochondrial function, meiosis, and chromosome organization pathways in oocytes. Consistent with these findings, mitotracker staining demonstrated improved mitochondrial distribution in oocytes following 630 nm treatment. Together, these results suggest that 630 nm LED phototherapy enhanced mitochondrial function, thereby significantly improving oocyte quality and embryo developmental potential, and potentially offering a novel strategy to enhance ART outcomes in ARA women.

To explore the translational potential of our preclinical findings, we conducted an initial clinical investigation using a customized wearable LED phototherapy device. This device employs flexible printed circuit boards integrated with silicone optical diffusers, delivering adjustable LED illumination in terms of wavelength, duration, and intensity, and can be comfortably secured around the abdomen (Figure 7). Given the mechanistic overlap between diminished ovarian reserve (DOR) and age‐related ovarian aging (ARA)—both characterized by reduced follicle number and impaired mitochondrial function—we selected DOR patients as a clinically relevant population for pilot testing. In this preliminary study, patients with DOR underwent daily 630 nm LED phototherapy for several days. We observed a significant improvement in AFC post‐treatment and a trend toward increased oocyte yield, suggesting that red‐light photobiomodulation may enhance ovarian reserve in humans, consistent with the functional recovery observed in the ARA mouse model. In establishing the phototherapy parameters, dosing differences between animal and human studies were considered. The mouse regimen (50 mW/cm^2^ for 5 min, 15 J/cm^2^ per day) was selected based on published PBM studies and further informed by pilot experiments.^44^ For clinical translation, adjustments were necessary due to the greater thickness and optical attenuation of human abdominal and pelvic tissues, which reduce the amount of red light reaching the ovary. Therefore, a lower irradiance of 15 mW/cm^2^ combined with a longer exposure of 30 min was used, resulting in a daily fluence of 27 J/cm^2^. This fluence is substantially lower than the maximum tolerated doses (320–480 J/cm^2^) reported in human red‐light safety trials, indicating that the selected parameters fall within an established safety range for initial clinical application.

A critical consideration for this transabdominal approach is whether sufficient 630 nm photons can penetrate the abdominal and pelvic wall to reach the ovaries. While direct intra‐ovarian measurement in patients is not feasible, our parameter selection is supported by a conservative theoretical estimation and the biological outcomes observed. Based on published optical properties of human tissue, the effective attenuation coefficient for 630 nm light in subcutaneous layers is estimated between 0.2 and 0.5 cm^−1.^ ^45^ For a typical path length of 3–6 cm in non‐obese women, application of the Beer–Lambert law (*I* = *I*₀ × e^(−*μ*_eff×*d*)^) suggests that ~10% of the surface irradiance (15 mW/cm^2^) could reach the ovarian region, yielding a local fluence on the order of 1.5 mW/cm^2^. This attenuated fluence remains within the range reported to elicit cellular photobiomodulation responses. Importantly, the significant increase in antral follicle count (AFC) observed post‐treatment provides indirect, clinical‐level support that a biologically effective signal was delivered to the ovarian environment. However, the clinical data remain limited. By the time of manuscript submission, only one patient achieved a spontaneous pregnancy following LED treatment. Continued follow‐up of the enrolled cohort is ongoing. In future, we are actively expanding patient recruitment, aiming to collect more robust datasets. These efforts will be critical to validate the therapeutic potential of LED phototherapy in women with DOR or ARA, and to inform evidence‐based clinical protocols.

While our findings are promising, this study has several limitations that warrant consideration. First, although we demonstrated the efficacy of specific LED parameters in our mouse model and initial clinical observation, the optimal photobiological parameters—such as wavelength selection, energy density, treatment duration, and timing relative to the menstrual or estrous cycle—remain to be systematically defined. Mechanistically, the precise photobiological mechanisms by which LED phototherapy influences SDHB expression and mitochondrial function, specifically whether these are direct cellular effects on oocytes or involve indirect regulatory pathways within the ovarian microenvironment, remain unclear. Moreover, the molecular mechanisms by which phototherapy exerts its effects in human ovarian tissue have yet to be elucidated. The specific chromophores absorbing the light energy and the subsequent signaling cascades also remain to be fully identified. Furthermore, while our preliminary clinical observation in a limited number of participants was encouraging, the long‐term safety and durability of treatment effects remain uncertain. Future work should include extended‐duration animal studies to assess potential long‐term or cumulative side effects associated with chronic LED phototherapy exposure before broader clinical application. Larger and well‐controlled longitudinal clinical trials will also be necessary to establish long‐term safety and efficacy in humans.

## 4 CONCLUSION

In summary, this study presents a non‐invasive approach to mitigating ovarian aging through mitochondrial‐targeted phototherapy, with the potential to improve both ovarian reserve and oocyte quality, and ultimately increasing pregnancy outcomes in ARA mice. Translating these findings, a wearable 630 nm LED device was developed and tested in a pilot clinical study involving women with DOR, where improvements in AFC and oocyte yield were observed. These results highlight the potential of 630 nm LED phototherapy as a safe, non‐invasive, and accessible strategy to enhance female fertility. Future large‐scale, controlled clinical trials are warranted to validate therapeutic efficacy and support broader clinical application in women of ARA or DOR.

## 5 EXPERIMENTAL SECTION

### 5.1 LED phototherapy device design and fabrication

The LED phototherapy device used for murine experiments was fabricated using chip‐on‐board packaging technology. LED chips emitting at peak wavelengths of 630 and 650 nm (red), and 850 and 950 nm (near‐infrared, NIR) were bonded onto ceramic substrates with thermally conductive silver adhesive, followed by baking, wire‐bonding, and encapsulation under protective glass covers. The LEDs were arranged in a circular array (9 × 37 chips) to ensure uniform irradiation. The device circuitry, configured with seven‐series and four‐parallel connections, operated at ~16 V and a current of 400 mA, regulated by a constant‐current LM3409 driver module (Texas Instruments) for stable intensity output. Spectral characteristics were verified by a spectrometer, and thermal stability was monitored using infrared imaging. To ensure accurate and reproducible dosing, irradiance (mW/cm^2^) was measured at the animal surface using an optical power meter. The device interface parameter does not directly represent irradiance; Table S1 provides the correspondence between interface values and measured power density at both the LED surface and after passing through the mouse restrainer. For all in vivo experiments, interface settings of 665 (630 nm) and 741 (850 nm) were used, corresponding to an actual irradiance of 50 mW/cm^2^ delivered to the ovarian region.

### 5.2 LED device temperature monitoring

The LED device was operated at a preset power density (50 mW/cm^2^) and was evaluated under simulated irradiation conditions. Surface temperatures of the LED device and the mounting fixture were continuously monitored using an infrared thermal imager. In parallel, the internal fixture temperature was recorded every 5 min using a thermometer for 30 min under both 630 and 850 nm illumination conditions.

### 5.3 Animals

Female wild‐type C57BL/6 mice were purchased from the Laboratory Animal Center of Peking University Health Science Center and housed under specific pathogen‐free (SPF) conditions. The animal study was conducted according to the Ethics Committee on Animals of Peking University People's Hospital (2023PHE024). For experiments, ARA mice (8 months old) and young control mice (2 months old) were used. After dorsal shaving near the ovarian region, mice were exposed daily to LED phototherapy for 15 consecutive days, approximately covering three estrous cycles. Following treatment, animals underwent mating experiments, ovarian histological analysis, oocyte quality assessment, and tissue sample collection as described below.

### 5.4 Hormone assays

Blood samples were collected retro‐orbitally from mice in all experimental groups during the estrous phase after the completion of the phototherapy regimen. Serum was separated overnight at 4°C, followed by centrifugation and stored at −80°C until analysis. Levels of follicle‐stimulating hormone (FSH), luteinizing hormone (LH), estradiol (E2), progesterone (P), and anti‐Müllerian hormone (AMH) were measured using standard radio immunoassays.

### 5.5 Mating and fertility assessment

At least six females per group were co‐caged with fertile wild‐type males at a female‐to‐male ratio of 2:1. The presence of a vaginal plug was checked daily to determine mating success. The pregnancy rate was calculated as the proportion of plug‐positive females that subsequently delivered offspring. Females showing plugs were isolated; those without plugs remained co‐caged for up to 3 weeks. The number of offspring per litter was also recorded for successful pregnancies.

### 5.6 Tissue dissociation and preparation

Freshly collected ovarian specimens underwent a triple rinse with Hank's Balanced Salt Solution (HBSS) and were subsequently minced into 1–2 mm fragments. These tissue portions were then subjected to enzymatic breakdown using 2 mL of GEXSCOPE Tissue Dissociation Solution (Singleron). The procedure was conducted at 37°C for 15 min in a 15 mL centrifuge tube with continuous agitation. Following the digestion step, the resulting cell suspension was filtered through sterile 40‐micron cell strainers. This filtrate was then pelleted by centrifugation at 1000 rpm for 5 min. The supernatant fluid was subsequently aspirated and discarded, and the cellular pellet was reconstituted in 1 mL of phosphate‐buffered saline (PBS; HyClone). To eliminate erythrocytes, 2 mL of GEXSCOPE red blood cell lysis buffer (Singleron) was added, and the mixture was maintained at 25°C for 10 min. Thereafter, the solution was centrifuged at 500*g* for 5 min, and the resulting cell pellet was resuspended in PBS. For viability assessment, cells were treated with 7‐AAD (1:1000 dilution; Invitrogen) on ice for a 30‐min period. Finally, live cells were isolated, and dead cells were excluded using flow cytometry.

### 5.7 Single‐cell RNA sequencing

Single‐cell suspensions containing 1 × 10^5^ cells each were prepared from processed ovarian tissue. For each group (young control, ARA, and 630 nm ARA), two biological replicates were processed separately, and each sample was labeled with CLindex barcodes (Singleron) before pooling. The pooled samples were then subjected to scRNA‐seq library preparation and sequencing. Single‐cell RNA sequencing libraries were constructed using the GEXSCOPE Single‐Cell RNA Library Kit (Singleron Biotechnologies) following the CLindex protocol. The mixed libraries were adjusted to a 4 nM concentration and consolidated into pools suitable for sequencing analysis. The combined pools were sequenced using an Illumina HiSeq X instrument. The instrumentation was configured to generate reads of 150 base pairs from each end.

### 5.8 scRNA‐seq quantifications and statistical analysis

Raw sequencing reads were aligned to the mm10 mouse genome reference. Subsequently, gene expression profiles were derived using an in‐house computational workflow. For subsequent analytical procedures on these RNA‐Sequencing outputs, the Seurat R package (v.4.1.0; http://satijalab.org/seurat/) was employed. The sequencing effort yielded an initial dataset comprising 12,244 cells, with contributions of 1668 from young control mice, 6596 from ARA mice, and 3980 from 630 nm ARA mice. A primary quality control filtration step was implemented to exclude cells exhibiting poor metrics. This was accomplished by establishing a criterion for mitochondrial gene content (relative to nuclear genes) below 10% and by ensuring the count of detected genes per cell ranged from 1000 to 4000. Consequently, a high‐quality dataset of 12,038 cells (young control mice: 1640; ARA mice: 6493; 630 nm ARA mice: 3905) was used for further computational analyses.

Data normalization was performed using Seurat's (v5.1.0) LogNormalize method, followed by identification of the top 2000 variable features for PCA. We used 30 principal components for PCA, and then applied Harmony (v1.2.1) batch correction to the PCA results to account for batch effects. The corrected low‐dimensional embeddings were used for SNN graph construction, UMAP visualization, and clustering (resolution = 0.8). Additionally, the current dataset was merged with the GSE106273 dataset employing Seurat's Fast Integration method, which utilizes reciprocal PCA.

### 5.9 DEGs identification, pathway enrichment

Differential gene expression testing was performed using the FindMarker function in the Seurat package with the Wilcox test. For inter‐group comparisons, only genes expressed in at least 10% of the cells in the case cells, with a maximum adjusted *p*‐value of 0.05 and a minimum absolute log2 fold change of 0.25, were considered significant DEGs. The top DEGs were selected for subsequent enrichment analysis. Gene ontology (GO) and KEGG pathway enrichment analyses were performed using the R package clusterProfiler (v4.12.6).

### 5.10 Smart‐seq RNA sequencing

Individual GV oocytes were manually collected into lysis buffer containing a ribonuclease inhibitor. Reverse transcription and full‐length cDNA amplification were performed using the Smart‐Seq2 protocol. Oligo(dT) primers were used for first‐strand synthesis, followed by PCR‐based cDNA amplification and magnetic bead purification. The quality and size distribution of amplified cDNA (~1–2 kb) were assessed using a Qubit 3.0 Fluorometer (Thermo Fisher Scientific) and an Agilent 2100 Bioanalyzer (Agilent Technologies). Purified cDNA was randomly fragmented, typically by ultrasonication, and sequencing libraries were prepared following the Illumina protocol, which includes DNA fragmentation, end repair, A‐tailing at the 3′ ends, adaptor ligation, PCR enrichment, and quality control. Library quality was evaluated using the LabChip GX Touch system (PerkinElmer) and StepOnePlus Real‐Time PCR System. Qualified libraries were then loaded on an Illumina HiSeq platform for PE150 sequencing. All library construction and sequencing procedures were carried out by Annoroad Gene Technology Co., Ltd. (Beijing, China).

### 5.11 Mitochondrial electron transport chain assays

Mitochondrial respiration was assessed in fresh ovarian tissue homogenates using the Oxygraph‐2k respirometer (Oroboros Instruments, Innsbruck, Austria). Fresh ovarian tissues were homogenized. Next, 100 μL aliquots of homogenate were analyzed using a substrate‐uncoupler‐inhibitor titration protocol involving the sequential addition of malate, pyruvate, glutamate, adenosine diphosphate (ADP), succinate, oligomycin, carbonyl cyanide‐*p*‐trifluoro‐methoxyphenyl hydrazone (FCCP), rotenone, antimycin A, ascorbate, and *N*,*N*,*N*′,*N*′‐tetramethyl‐*p*‐phenylenediamine dihydrochloride (TMPD). Respiratory parameters (CI leak, CI OXPHOS, Complex I + II OXPHOS, ATP synthesis, maximum ETS capacity, Complex II ETS, residual oxygen consumption, and Complex IV activity) were recorded and analyzed using DatLab software (v7.4). Separately, intracellular ATP levels in isolated oocytes and granulosa cells were quantified using an ATP luminescence assay kit (Luminescence assay; Beyotime, Cat no. S0026). For ATP measurements, GV or MII oocytes were collected and pooled for each biological replicate (typically 8–15 oocytes per pool). ATP was extracted according to the manufacturer's protocol, and the total luminescence value was normalized to the number of oocytes in the pool, yielding ATP content per oocyte. ATP levels are reported as relative luminescence units per oocyte.

### 5.12 Paraffin embedding and immunohistochemistry

Ovarian tissues were fixed in 4% paraformaldehyde for 24 h, dehydrated through a graded ethanol series, embedded in paraffin wax, and sectioned at 5 μm thickness. For immunohistochemical staining, sections underwent deparaffinization, rehydration, and antigen retrieval using a standard heat‐induced epitope retrieval (HIER) citrate buffer (pH 6.0). Endogenous peroxidase activity was quenched with 3% hydrogen peroxide in methanol for 10 min, followed by blocking of nonspecific binding with 5% normal goat serum. Sections were incubated overnight at 4°C with primary antibody against Ki67 (Abcam, ab15580, 1:200). The following day, samples were incubated with an appropriate HRP‐conjugated secondary antibody, visualized using DAB substrate, counterstained with hematoxylin, and dehydrated. The slides were observed and photographed with an Olympus BX51 microscope and an Olympus DP73 CCD photographic system.

### 5.13 TUNEL staining

Apoptotic cells in ovarian tissue sections were detected using a TUNEL assay kit according to the manufacturer's instructions (Roche Applied Science). Briefly, after standard deparaffinization and rehydration, sections were permeabilized by incubation with proteinase K for 15 min. Sections were subsequently incubated with the TUNEL reaction mixture at 37°C for 60 min. Slides were counterstained with hematoxylin. The slides were observed and photographed with an Olympus BX51 microscope and an Olympus DP73 CCD photographic system.

### 5.14 Western blotting

Ovarian tissues were lysed in RIPA supplemented with protease and phosphatase inhibitors (Roche), followed by centrifugation at 12,000 rpm for 15 min at 4°C. Protein concentrations were determined using the BCA assay. Equal amounts of protein (30 μg per lane) were separated by SDS–PAGE and transferred to PVDF membranes (Millipore). Membranes were blocked with 5% non‐fat dry skim milk in TBST at room temperature for 1 h and then incubated overnight at 4°C with primary antibodies against SDHB (1/1000 dilution; Abmart, T56860), ATP5A1 (1/1000 dilution; Abmart, T56775), cytochrome c (Cyt‐C) (1/1000 dilution; Abmart, TA5229), VDAC1 (1/1000 dilution; Abmart, T55416), and GAPDH (1/5000 dilution; Proteintech, 60004‐1‐Ig). After washing, membranes were incubated with HRP‐conjugated secondary antibodies “Implied pairings based on primary host: HRP‐conjugated goat anti‐rabbit IgG (ZSGB‐BIO, ZB‐2301) and HRP‐conjugated goat anti‐mouse IgG (ZSGB‐BIO, ZB‐2305),” diluted 1:5000 in blocking buffer. Signals were detected using enhanced chemiluminescence (ECL) reagents (Thermo Fisher Scientific).

### 5.15 Quantitative real‐time PCR (qPCR)

Total RNA was extracted from mouse ovarian tissues using TRIzol reagent (Invitrogen, USA) according to the manufacturer's instructions. First‐strand cDNA synthesis was performed using 1 μg of total RNA with the PrimeScript RT reagent kit (Takara RR047A, Japan). Quantitative PCR was conducted using SYBR Green Master Mix (Takara RR420A, Japan). *Gapdh* was used as the internal reference gene, and relative gene expression levels were calculated using the 2–ΔΔCt method. Primer sequences are listed below:

*Rec8*: F TATGTGCTGGTAAGAGTGCAAC, R TGTCTTCCACAAGGTACTGGC
*Tdrd5*: F ACAGAACTTGTTGGCGCTCT, R ATGAAAGCGGTCCACCAGAC
*Pld6*: F GGGTCATCACTGACTGCGAC, R CGTACCTGTATCCCTGCCTTG.
*Inhbb*: F TCAGCTTTGCAGAGACAGATGG, R ACACCTTGACCCGTACCTTC.
*RYR3*: F GGACCAGGAACGGAAGAAGAC, R ACTTCTTCATCAGTGTCCCTACAG

### 5.16 Oocyte collection and maturation

Female mice were superstimulated with an intraperitoneal injection of 5 IU pregnant mare's serum gonadotropin (PMSG) to collect the GV oocytes. Female mice were sacrificed by cervical dislocation 48 h after PMSG injection and GV oocytes were collected and washed thoroughly to remove cumulus cells by genital pipetting. Oocytes retrieved were cultured in a small drop of M16 (M7292; Sigma‐Aldrich) by maintaining 5% CO_2_ at 37 °C for 18 h. Oocyte maturation was assessed by determining the proportion of oocytes that progressed to the MII stage.

### 5.17 In vitro fertilization (IVF) assays

Female mice received sequential injections (5 IU PMSG followed by 5 IU human chorionic gonadotropin (hCG), 46 h interval). Oocytes were harvested 13–15 h post‐hCG injection, co‐incubated with capacitated sperm (37°C, 6 h), and cultured. Fertilization (2‐cell rate at 24 h) and embryonic developmental competence (4‐cell at 48 h, 8‐cell at 72 h, and morula/blastocyst at 96 h) were assessed microscopically.

### 5.18 Visualization of the mitochondria by MitoTracker green

Mitochondrial distribution was evaluated by incubating live cells with Mitotracker (1/2500, Invitrogen, M7514) for 30 min at 37°C. After washing, mitochondrial distribution patterns were analyzed under confocal microscopy.

### 5.19 Clinical study design and participants

This prospective, single‐arm interventional study was conducted at Peking University People's Hospital between May 2024 and April 2025 (Ethics approval number: 2023PHB252‐001). The objective was to evaluate the feasibility and efficacy of wearable 630 nm LED phototherapy in improving ovarian reserve parameters among women with diminished ovarian reserve (DOR) undergoing IVF/ICSI. All participants provided written informed consent prior to enrollment, in accordance with the Declaration of Helsinki. As an early exploratory investigation, the trial was not registered.

Eligible participants were women aged 20–45 years who met the Bologna criteria for DOR. Inclusion required at least two of the following: advanced age (≥40 years) or risk factors for poor ovarian response (POR); a history of POR (≤3 oocytes retrieved in a previous IVF cycle); antral follicle count (AFC) <7 or anti‐Müllerian hormone (AMH) <1.1 ng/mL. Exclusion criteria included severe adenomyosis, endometriosis, submucosal fibroids, endometrial malignancy or premalignant lesions, and significant intrauterine adhesions.

A total of 10 patients were enrolled. After signing informed consent, each participant visited the clinic on day 2 of their menstrual cycle for baseline hormone tests and transvaginal ultrasound. The LED device was dispensed and usage instructions were provided. Phototherapy began on the same day, with patients applying the device to the lower abdomen daily for 30 min. The device emitted continuous 630 nm red light at 15 mW/cm^2^ and was used until the day before oocyte retrieval.

### 5.20 Clinical IVF/ICSI protocol

A comprehensive infertility evaluation was conducted for patients referred to the reproductive center. Individualized controlled ovarian stimulation was administered based on physicians' experiences. The details of the stimulation protocols and oocyte retrieval procedures have been previously described.

### 5.21 Outcome measures

The primary outcome was the change in AFC before and after LED phototherapy. The secondary outcome was the change in the number of oocytes retrieved during the subsequent IVF/ICSI cycle. All outcomes were evaluated within 3 months after completion of the intervention. Detailed clinical outcomes, including spontaneous pregnancy events, were recorded during follow‐up.

### 5.22 Statistical analysis for preclinical studies

The data are presented as mean ± SEM unless otherwise specified. Comparisons between two groups were performed using a two‐tailed unpaired Student's *t*‐test. For comparisons involving three or more groups, one‐way ANOVA followed by Tukey's post hoc test was applied. All statistical analyses were conducted using GraphPad Prism 10 (GraphPad Software, San Diego, CA). A *p* value <0.05 was considered statistically significant. Statistical significance is indicated as follows: *p* < 0.05 (*), *p* < 0.01 (**), *p* < 0.001 (***).

### 5.23 Statistical analysis for clinical study

Continuous variables were expressed as median (interquartile range, IQR) and compared using the Mann–Whitney *U* test. Categorical variables were expressed as counts (percentages). A two‐sided *p*‐value <0.05 was considered statistically significant. Statistical analyses were performed using SPSS 26.0 (SPSS, Chicago, IL).

## AUTHOR CONTRIBUTIONS

J.W. and L.T. designed the study and provided their valuable contributions to the whole study. T.S and Y.W performed the experiments. F.F. and Z.L. were responsible for coordinating the enrolment and management of female participants. C.C., J.A. and N.H. assisted with clinical volunteer management. R.L., J.S. and X.L. critically reviewed the paper. J.X. provided the LED devices. All authors read and approved the final manuscript.

## CONFLICT OF INTEREST STATEMENT

The authors declare no competing interests.

## Supporting information


**Figure S1.** Overview of the representative participant's ART course, LED phototherapy, and pregnancy outcome. (A) Timeline of ART and 630 nm LED phototherapy for the enrolled participant (This figure was created in BioRender. W, L. (2026) https://BioRender.com/jy6xy0z). (B) Overview of 11 ART cycles performed pre‐ and post‐LED phototherapy in the participant. (C) Oocyte retrieval and high‐quality embryo development from IVF Cycle 6 in the pertinent, following a period of dual‐wavelength LED phototherapy. (D) Representative ultrasound images from the participant following spontaneous natural pregnancy. Left to right: 6 + 4 weeks (embryonic bud), 6 + 4 weeks (embryonic bud), and 12 + 4 weeks (fetus).
**Figure S2.** Relative proportions of annotated ovarian cell types in young control, untreated ARA, and 630 nm LED–treated ARA mice, based on single‐cell RNA‐seq data.
**Table S1.** Measured irradiance at the LED surface and after passing through the mouse fixation device for 630 and 850 nm sources.

## Data Availability

The single‐cell RNA‐seq data generated in this study have been deposited in the NCBI Sequence Read Archive (SRA) under BioProject accession PRJNA1390951.
