# Supplementary material for: 630 nm LED phototherapy enhances ovarian function and fertility potential in advanced reproductive age females
Source: Bioeng Transl Med. 2026 Feb 20;11(3):e70117. doi: 10.1002/btm2.70117 (PMC13247421; doi:10.1002/btm2.70117)
Supplement: Supplementary file 1 — Figure S1. Overview of the representative participant's ART course, LED phototherapy, and pregnancy outcome. (A) Timeline of ART and 630 nm LED phototherapy for the enrolled participant (This figure was created in BioRender. W, L. (2026) https://BioRender.com/jy6xy0z). (B) Overview of 11 ART cycles performed pre‐ and post‐LED phototherapy in the participant. (C) Oocyte retrieval and high‐quality embryo development from IVF Cycle 6 in the pertinent, following a period of dual‐wavelength LED phototherapy. (D) Representative ultrasound images from the participant following spontaneous natural pregnancy. Left to right: 6 + 4 weeks (embryonic bud), 6 + 4 weeks (embryonic bud), and 12 + 4 weeks (fetus). Figure S2. Relative proportions of annotated ovarian cell types in young control, untreated ARA, and 630 nm LED–treated ARA mice, based on single‐cell RNA‐seq data. Table S1. Measured irradiance at the LED surface and after passing through the mouse fixation device for 630 and 850 nm sources. [file BTM2-11-e70117-s001.docx]

**Table of Contents**

**630 nm LED Phototherapy Enhances Ovarian Function and Fertility Potential in Advanced Reproductive Age Females**

**Supporting Information**

**630 nm LED Phototherapy Enhances Ovarian Function and Fertility Potential in Advanced Reproductive Age Females**

*Tiantian Su,* *Yanling Wan,* *Fang Fang,* *Ziyu Li,* *Cheng Cheng,* *Jiajia Ai,* *Nannan Huang,* *Rong Liang,* *Jingrong Song,* *Xiaowei Li,* *Jiangen Xu,* *Jianliu Wang,** *Li Tian**

**
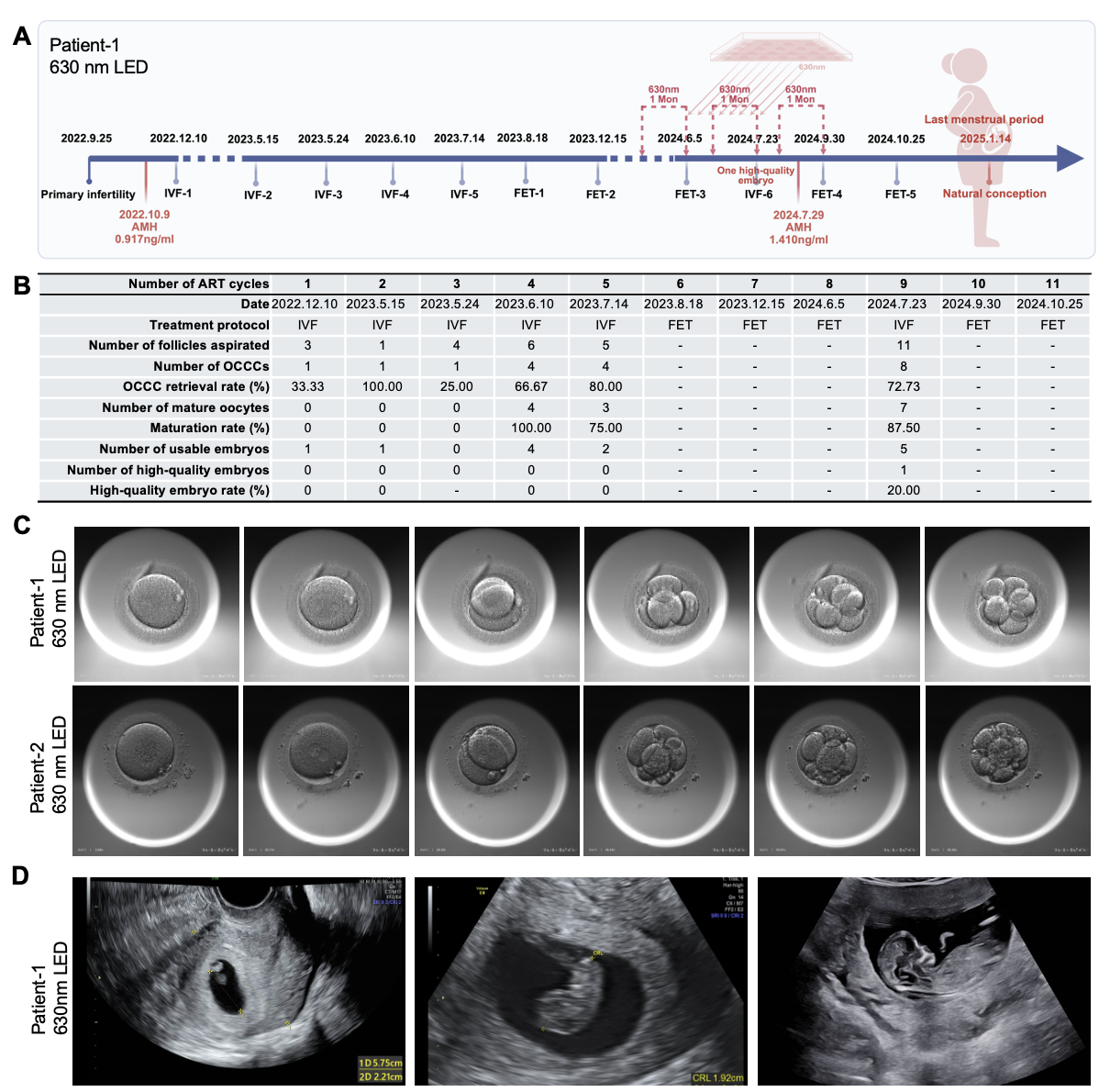
**
Figure S1. Overview of the representative participant’s ART course, LED phototherapy, and pregnancy outcome. (A) Timeline of ART and 630 nm LED phototherapy for the enrolled participant (This figure was created in BioRender. W, L. (2026) <https://BioRender.com/jy6xy0z>). (B) Overview of 11 ART cycles performed pre- and post-LED phototherapy in the participant. (C) Oocyte retrieval and high-quality embryo development from IVF Cycle 6 in the pertinent, following a period of dual-wavelength LED phototherapy. (D) Representative ultrasound images from the participant following spontaneous natural pregnancy. Left to right: 6+4 weeks (embryonic bud), 6+4 weeks (embryonic bud), and 12+4 weeks (foetus).

| **Target irradiance (mW/cm²)** | **630 nm** | | **850 nm** | |
| --- | --- | --- | --- | --- |
|  | **LED surface** | **After fixation device** | **LED surface** | **After fixation device** |
| 5 mW/cm^2^ | 67 | 86 | 1 | 1 |
| 10 mW/cm^2^ | 130 | 162 | 132 | 160 |
| 15 mW/cm^2^ | 180 | 230 | 190 | 236 |
| 20 mW/cm^2^ | 228 | 292 | 241 | 309 |
| 25 mW/cm^2^ | 271 | 348 | 293 | 376 |
| 30 mW/cm^2^ | 312 | 406 | 342 | 445 |
| 35 mW/cm^2^ | 352 | 466 | 384 | 516 |
| 40 mW/cm^2^ | 393 | 527 | 440 | 587 |
| 45 mW/cm^2^ | 434 | 594 | 485 | 661 |
| 50 mW/cm^2^ | 483 | 665 | 533 | 741 |
| 55 mW/cm^2^ | 531 | 733 | 587 | 817 |
| 60 mW/cm^2^ | 579 | 801 | 630 | 892 |

Table S1. Measured irradiance at the LED surface and after passing through the mouse fixation device for 630 nm and 850 nm sources.


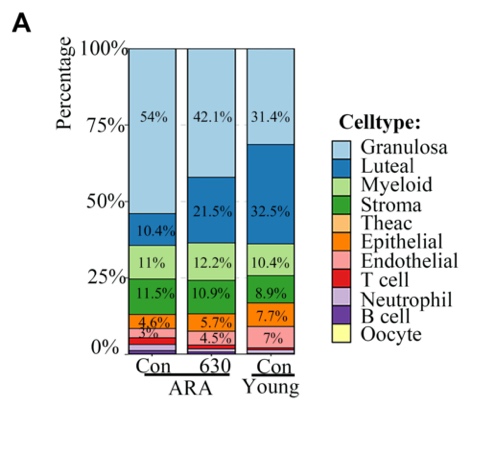


Figure S2. Relative proportions of annotated ovarian cell types in young control, untreated ARA, and 630 nm LED–treated ARA mice, based on single-cell RNA-seq data.
